# Dual Modality Noncontact Photoacoustic and Spectral Domain OCT Imaging

**DOI:** 10.1177/0161734615582003

**Published:** 2016-01

**Authors:** Elisabeth Leiss-Holzinger, Johannes Bauer-Marschallinger, Armin Hochreiner, Philipp Hollinger, Thomas Berer

**Affiliations:** 1 Research Center for Non-Destructive Testing GmbH (RECENDT), Linz, Austria

**Keywords:** photoacoustic imaging, optical coherence tomography, galvanometer scanning, remote sensing, interferometry

## Abstract

We developed a multimodal imaging system, combining noncontact photoacoustic imaging and optical coherence tomography (OCT). Photoacoustic signals are recorded without contact to the specimens’ surface by using an interferometric technique. The interferometer is realized within a fiber-optic network using a fiber laser at 1550 nm as source. The fiber-optic network allows the integration of a fiber-based OCT system operating at a wavelength region around 1310 nm. Light from the fiber laser and the OCT source are multiplexed into one fiber using wavelength-division multiplexing. The same focusing optics is used for both modalities. Back-reflected light from the sample is demultiplexed and guided to the respective imaging systems. As the same optical components are used for OCT and photoacoustic imaging, the obtained images are co-registered intrinsically in lateral direction. Three-dimensional imaging is implemented by hybrid galvanometer and mechanical scanning. To allow fast B-scan measurements, scanning of the interrogation beam along one dimension is executed by a galvanometer scanner. Slow-axis scanning, perpendicular to the fast axis, is performed utilizing a linear translational stage. We demonstrate two-dimensional and three-dimensional imaging on agarose phantoms.

## Introduction

In photoacoustic imaging (PAI), a semitransparent sample is illuminated by short pulses of electromagnetic radiation, most commonly by short laser pulses. The locally varying specific absorption and thermal properties lead to a locally varying temperature rise. The temperature rise leads to thermal expansion, and finally, to the generation of broadband ultrasonic waves. By recording these ultrasonic waves, the initial pressure distribution can be assessed by using dedicated reconstruction algorithms. Thus, PAI is a hybrid technique making use of optical absorption and ultrasonic wave propagation. For recording of the ultrasonic waves, usually piezoelectric transducers are used. Although, without doubt, these transducers are perfectly suited for many applications, they exhibit several drawbacks. First, these transducers are usually opaque. This leads to a shadowing effect in the so-called backward mode, where illumination/excitation is done from the very side where the detector is located. Second, high-resolution imaging requires the transducer to be smaller than the smallest structure to be imaged. Reducing the dimension of the transducer, however, leads to deterioration of the signal/noise ratio (SNR).^1^ This problem could be overcome by using focused transducers, such as those used in acoustic-resolution photoacoustic microscopy. However, for focused transducers, the region of high spatial resolution is limited to a depth range that coincides with the nondivergent region of the transducer focus.^2^ Third, piezoelectric transducers are sensitive only around a center frequency, which can lead to a decreased image quality. Fourth, as the most severe drawback, there are applications where contacting means are prohibited. This is the case for many in-line material inspection applications as well as for certain kind of surgeries, such as brain surgeries.^3^ For burn diagnostics contacting means should be avoided^4^ as well. To a certain extent, the mentioned drawbacks can be overcome by using optical detection techniques.^1,2,5-11^ However, only noncontact PAI (ncPAI) techniques^4,12-19^ can solve all of these problems.

In ncPAI, interferometric means are used to measure displacements on a sample surface, caused by the impinging ultrasonic waves, without requiring contact with the sample. Instead of the opaque transducer, an interrogation laser beam is used that does not block the excitation light. The laser beam can be focused onto a small spot, without decreasing the SNR. By using appropriate interferometers, the transmission characteristics can range from a few Hertz up to several hundreds of Megahertz. For Michelson or Mach-Zehnder interferometers, the upper detection frequencies are only limited by the bandwidths of the used photo receivers. Recently, we demonstrated ncPAI using a fiber-based interferometer.^20^ The realization of ncPAI in a fiber-optic network allows the straightforward combination with other fiber-based imaging modalities. In this paper, we present the combination with fiber-based optical coherence tomography (OCT).

OCT is a high-resolution imaging method that allows the acquisition of depth resolved images of (sub)surface features. OCT employs the partial coherence properties of a broadband light source and interferometry to locate the positions of reflective and backscattering interfaces.^21,22^ This technique was initially developed for ophthalmology, but is meanwhile used in various other biomedical applications and also for material inspection.^23^ As OCT is a noncontact method, it is ideally suited as an interoperative imaging tool. OCT has already been used during the surgery of laryngeal carcinoma^24^ and neurosurgery of the human cortex.^25^ Combinations of PAI and OCT have been demonstrated before.^16,26-32^ However, with exception of the work of Wang et al.,^16^ these methods relied on contacting transducers. In Wang et al.,^16^ the same low-coherence light source was used for OCT and noncontact PAI. However, an oil film was needed for coherence gating and for providing a smooth surface. Thus, these approaches did not make full use of the remote nature of OCT. This limitation could be overcome by using noncontact PAI techniques instead of the contacting transducers.^33,34^ In this paper, we present multimodal ncPAI and OCT imaging, based on Berer et al.^33,34^ The light sources for fiber-based ncPAI and for spectral domain OCT (SD-OCT) are multiplexed into one measurement head. As the same fiber network and same optical components are used for photoacoustic and OCT imaging, the obtained images are co-registered intrinsically in lateral direction. For fast PAI, we introduce scanning by a galvanometer mirror.

The paper is constructed as follows. The “Setup” section is dedicated to the description of the optical setup and the electronic setup. In the “Measurements” section, two-dimensional (2D) and three-dimensional (3D) imaging on agarose phantoms is demonstrated. In the “Discussion and Outlook” section, we present a discussion of the results and give an outlook to future works.

## Setup

### Optical Setup: Remote PAI and OCT

**Figure 1.**
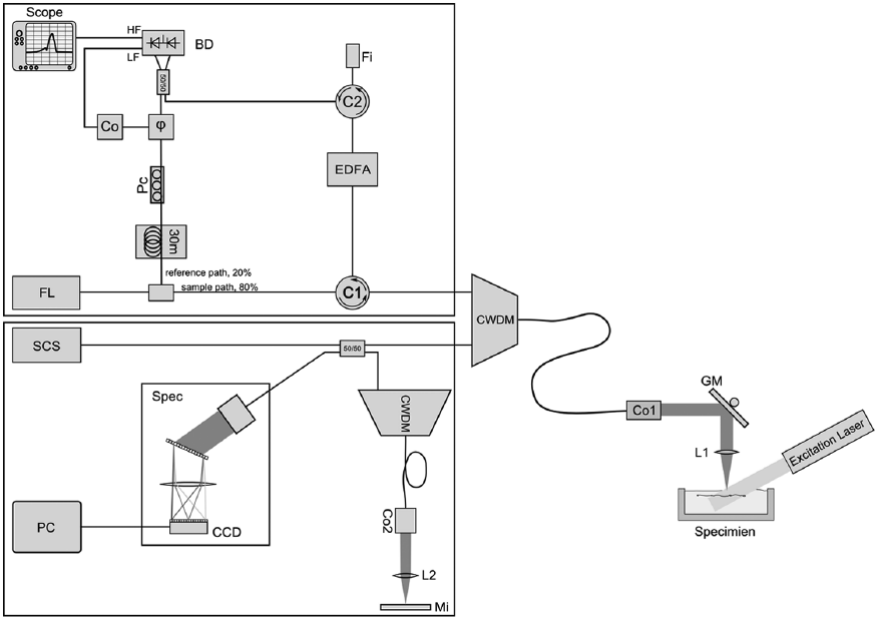
Schematic of the optical setup.

A schematic of the optical setup is depicted in Figure 1. The setup consists of two parts, namely, the setups for ncPAI imaging and for SD-OCT. The ncPAI and OCT systems use different light sources, that is, a light source at 1550 nm and a broadband light source between 1200 and 1600 nm. For combination of the two wavelength regions, a coarse wavelength-division multiplexer (CWDM) is used. The CWDM allows bidirectional operation to combine and split the two wavelength bands.

For ncPAI, a small bandwidth continuous wave fiber laser (FL, Koheras AdjustiK) is used as source for the interferometer. The laser has a central wavelength of 1549.9 nm that can be tuned in the range of ±250 pm by thermal tuning; the laser line width is below 3 kHz, and the maximum output power is 25 mW. Laser light is coupled into a SMF-28e+ single mode fiber (SMF), which is the only type of fiber used in the setup. The light is divided into a fiber-guided reference beam and a fiber-guided sample beam by an 80/20 splitter. The major part, that is, 80% of the light, is directed toward the sample surface via a circulator (C1) and the wavelength-division multiplexer. A collimator (Co1) converts the light from the fiber into a free-space collimated beam, which is focused onto the sample surface by an achromatic lens (L1) with a focal length of 75 mm. The lateral position of the focal point can be changed one-dimensionally by a galvanometer scanner (GM, Cambridge Technology). Light that is reflected from the sample surface is collected by L1 and coupled into the fiber via GM and Co1. The power of the collected light is usually low, typically only 0.1% of the incident light power. To increase the power level, the light is directed to an erbium-doped fiber amplifier (EDFA, Ericsson PGE-608-30-PA) via C1, where the light is amplified by optical amplification. The EDFA accepts input signals in the range of −40 dBm to −10 dBm; the nominal amplification at −30 dBm is 28 dB. With more than 30 dB for power levels between 0.5 µW and 8 µW, the amplification was found to be slightly higher. EDFAs exhibit broadband spectral noise caused by amplified spontaneous emission (ASE). The ASE noise spectrum is approximately the same as the gain spectrum of the amplifier, which is between 1528 nm and 1563 nm. For reduction of ASE, a small spectral bandwidth filter (Fi) with a spectral width of 0.25 nm is used in a reflection configuration. The center frequency of Fi is 1550.12 nm; the wavelength of the source laser is thermally tuned to match this wavelength. A circulator directs the filtered light toward a balanced photodetector (BD) via a −3dB coupler, where the light is recombined with the light of the reference arm. Phase instabilities of the laser cause phase noise in the interference signal if the lengths of the reference and sample arms are not matched. Therefore, the reference arm is matched to the sample arm using a fiber coil with a length of about 30 m, which compensates the length of the erbium-doped fiber in the EDFA. To match the polarization states of the reference and sample arms, a three-paddle polarization controller (Pc) is used. The BD features two outputs for low and high frequencies. At the high-frequency (HF) output, the ultrasonic signals are measured with a digital scope (LeCroy WaveRunner 44Xi-A). The low-frequency (LF) output is used for phase stabilization of the interferometer. The interferometer is stabilized at the quadrature point using an electro-optical phase shifter (ϕ) and a digital controller (Co). The digital controller and the photodetector are described in detail in the next section. Photoacoustic waves within a sample are excited using a frequency-doubled Q-switched Nd:YAG laser (Continuum Surelite SL I-20) at a wavelength of 532 nm. The laser has a repetition rate of 20Hz, a maximum output energy of 160 mJ/pulse, and a pulse width of 4 to 6 ns.

As light source for SD-OCT, the high wavelength output of a pulsed supercontinuum source (SCS, NKT Koheras SuperK Versa) is used. The output covers a wavelength region from 1200 to 1900 nm, but because of the CWDM, the effective wavelength range is reduced to Δλ = 100 nm, which is the transmission window at λ_0_ = 1310 nm. The physical limit for the axial resolution, given as is therefore 7.5 µm.^34^ Light from the light source is coupled into the CWDM with a −3 dB coupler. In the CWDM, the narrowband light at 1550 nm from the ncPAI system is combined with the OCT broadband light. The combined light is then focused onto the sample surface as described above. Light that is backscattered is collected and guided to CWDM, where the two wavelength regions for ncPAI and OCT are separated. The reference arm of the OCT consists of a second CWDM, a collimator Co2, a lens L2, and a mirror Mi. A free-beam path is used to match the path lengths of sample and reference arm. To minimize dispersion, lens L2 and collimator Co2 are of the same type as used in the sample arm. In addition, a CWDM of the same model as in the sample arm is included to compensate for its dispersion. Additional dispersion, caused, for example, by production-related tolerances of the CWDMs is compensated numerically during data processing. Light from sample and reference arm are interfered on a spectrometer (Spec). The spectrometer consists of a high-speed InGaAs line scan camera (CCD, Sensors Unlimited GL2048), lens L3, and a volume phase holographic transmission grating (Gr). The CCD features 2048 pixels with a pitch of 10 µm, an aperture of 210 µm, and a digital resolution of 12 bit. Data from the CCD are transmitted via camera link to a frame grabber card inside a standard PC. The maximum rate of the CCD is 76,000 lines/s. The lens L3 has a focal length of 62 mm, and the transmission grating features 1145 lines/mm.

$$\frac{2\cdot \ln2}{\pi }\cdot \frac{\lambda_{0}^{2}}{\Delta \lambda },$$

### Electronic Setup: Detector and Controller

**Figure 2.**
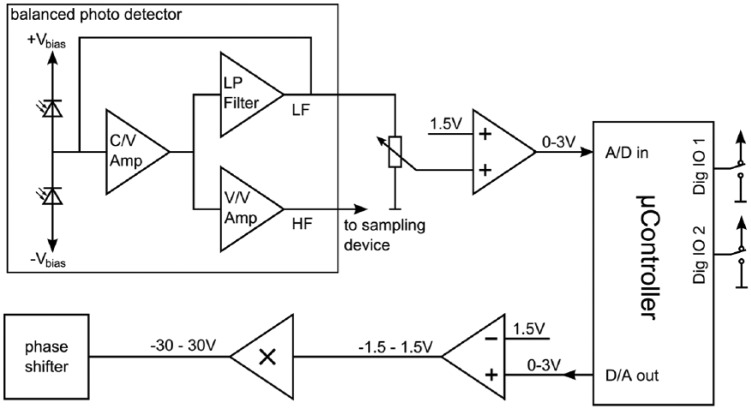
Schematics of the electronics.

The photodetector and digital controller are schematically depicted in Figure 2. For converting the optical encoded ultrasonic signals to electric signals, a self-made balanced photodetector^35^ is used. The photocurrent of the reversed biased photodiodes is converted into a voltage by the transimpedance amplifier (C/V Amp). A second stage (V/V) is used to amplify this voltage with a gain of about 100 dB. Such a high-gain amplification can lead to saturation of the amplifier if the working point of the interferometer is not maintained precisely, which is why the photodiodes are not illuminated by the same light intensity. To prevent the amplifier from saturating, an active low-pass filter feedback loop is used to remove LF disturbances. The feedback loop allows a large bandwidth, as no additional capacitive load is used, whereas passive filtering using a band-pass filter would reduce the allowed bandwidth. The output of the low-pass filter is tapped and used for stabilization of the operating point. The HF gain of the detector has been measured to be about 100 dB between 1 and 10 MHz; the −3 dB bandwidths are 100 kHz and 45 MHz. When terminated with 50 Ohm, the output voltages can range between −2.2 and +2.2 V.

For stabilization of the operating point, a digital controller based on an Arduino Due platform is used. The Arduino Due features an ARM Cortex-M3 processor at a clock of 84 MHz and offers several 12-bit digital–analog converters (DAC), 12 bit analog–digital converters (ADC), and 1-bit digital input/output (IO) ports. The LF output of the photodetector is sampled by an ADC port (A/D in). As the used port allows only for a voltage between 0 and 3 V, the symmetric output voltage of the photodetector is first reduced by a potentiometer and then raised by 1.5 V using a summing amplifier. A PID-controller program is implemented on the microcontroller and maintains the operating point of the interferometer. The operating point is maintained by setting the voltage of the phase shifter in such a way that the LF output of the photodetector is zero. For phase-shifting, an un-terminated electro-optical phase shifter (Thorlabs LN68S-FC) is used. The phase shifter allows phase-shifts slightly larger than ±6π corresponding to an applied voltage of ±30 V. The time-dependent control voltage of the phase shifter is determined by a 12-bit output channel (D/A out) of the microcontroller, with an output voltage of 0 to 3 V. To obtain a symmetric voltage, the output voltage is reduced by 1.5 V using a second summing amplifier. Subsequently, the voltage is amplified 20-fold by a voltage amplifier. Two switches connected to digital IO ports (Dig IO 1, 2) are used to set different modes of the controller.

Because of the long fiber optical network, small temperature drifts can lead to rather large changes in the optical path length. In addition, dynamic changes in the length of the sample arm larger than 4.5 µm occur during scanning, if the sample surface is not ideally flat or slightly tilted. As a consequence, the phase shift region of ±6π is often not sufficient to stabilize the operating point of the interferometer. Thus, whenever the phase shifter is on its limit, that is, if ±6π is reached, a change of the operating point of 10π in the opposite direction is introduced. As the operating point is symmetric in n⋅2π, with *n* being a natural number, the operation point is maintained.

### Scanning

Fast-axis scanning uses the galvanometer scanning mirror for deflection of the interrogation beam. For scanning we use a function generator (Agilent 33250A) to control the scanning mirror via a driver that converts the output voltage to a current. The output function is a ramp. Whenever the ramp is started, a trigger signal triggers the frame grabber card of the OCT system and a new frame is recorded. Photoacoustic signals, acquired with the digital scope, are recorded at each pulse of the excitation laser without averaging. Slow-axis scanning, perpendicular to the fast axis, is accomplished by a motorized linear translational stage (Physik Instrumente M-413.22S). In the present setup, the linear stage is not synchronized to the rest of the system and is controlled by the operator.

### Photoacoustic Reconstruction

After acquisition of the ultrasonic data, an image of the optical absorption is reconstructed. For reconstruction, we use a Fourier domain synthetic aperture focusing technique (FSAFT).^15,36^ This algorithm is commonly used for pulse-echo measurements, but can also be used for single transmission measurements, as in our case. In the first case, the sound velocity for the reconstruction is set to half of the velocity in the medium; in the latter (our) case, simply the sound velocity has to be used.

## Measurements

### Sample Preparation

All presented measurements were performed on biological samples embedded into agarose. Agarose gel was prepared by dissolving agarose in hot water with a content of 1 g agarose per 50 ml water. A drop of milk was mixed into the agarose to mimic the scattering properties of tissue. Agarose was then filled into a Petri dish and the biological samples were immersed. After cooling, the agarose/milk phantoms could be used for imaging.

### Measurement Procedure

Photoacoustic signals were generated with pulses from the frequency-doubled Nd:YAG laser at a pulse energy of 20 mJ. The excitation spot was of elliptical shape with the lengths of the major and minor axes being about 10 mm and 7 mm, respectively. The resulting fluence of 35 mJ/cm^2^ was about two times the allowed MPE (maximum permissible exposure) for skin at 532 nm. Because of the low repetition rate of the excitation laser of 20 Hz, OCT is orders of magnitudes faster than ncPAI. Therefore, regions of interest were first located by OCT and subsequently measured by ncPAI. OCT measurements were performed at a rate of 6150 lines/s. The time required for one B-scan image was below 170 ms. The ncPAI data were acquired at a rate of 20 Hz and the data acquisitions over 150 points required 7.5 s. The scanning distance was 7 mm and was constant for all measurements. Slow-axis scanning was performed with the translational stage. After acquisition of the OCT and ncPAI B-scan data, the measurement was stopped and the stage was moved to a new position. Subsequently, data acquisition was started again.

### Three-Dimensional Measurement of a Hair/Agarose Phantom

**Figure 3.**
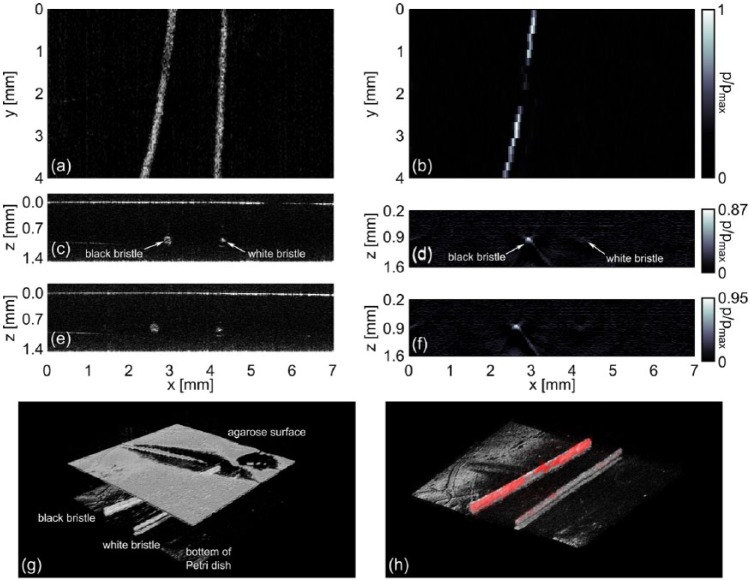
OCT measurement and photoacoustic reconstruction of a hair/agarose phantom: (a) en-face representation of the OCT data; (b) MIP along *z*-direction of the photoacoustic reconstruction; (c) OCT B-scan image at *y* = 1 mm; (d) Photoacoustic section image in the *x*-*z* plane at *y* = 1 mm; (e) and (f) OCT B-scan and photoacoustic section image at *y* = 3 mm; (g) 3D representation of the OCT measurement; (h) 3D representation of the OCT measurement and the PAI reconstruction. The agarose surface is removed for illustrative reasons. OCT = optical coherence tomography; MIP = maximum intensity projection; PAI = photoacoustic imaging.

Several black and white bristles, with diameters of approximately 150 µm, were embedded into agarose as described in section “Sample Preparation.” A region with one black and one white bristle was identified by OCT. Data acquisition was performed as described above. 21 ncPAI B-scans were acquired over an area of 7 mm × 4 mm, leading to a distance of 200 µm between the individual scans. Each photoacoustic B-scan consisted of 150 measurement points, resulting in a distance of approximately 47 µm between the points. For OCT, B-scans were acquired at 101 *y*-positions with steps of 40 µm between the scans. Each scan contained 1040 A-lines, resulting in a distance of 7 µm between individual A-lines. The en-face representation of the OCT data in Figure 3(a) shows the black and the white bristle. The maximum intensity projection (MIP) of the ncPAI reconstruction along *z*-direction in Figure 3(b) shows only the black bristle. The white bristle cannot be identified, as only little photoacoustic signals had been generated because of low absorption. A slight colormap shift was applied to the data to enhance contrast. The corresponding colorbar is presented right of the image. In the region around *y* = 2 mm, the hair seems to be broken. The reason can be seen from the 3D-rendering of the OCT data in Figure 3(g). In the region around *y* = 2 mm, backscattering from the surface into the measurement head was low, possibly because of ripples on the high-reflecting sample surface. Consequently, only weak ncPAI signals were recorded in this region. Figures 3(c) to (f), show ncPAI and OCT B-scan images in the *x*-*z* plane at *y* = 1 mm and 3.0 mm. Also here, both bristles can easily be identified for OCT. In the ncPAI image, the black bristle is clearly visible. Also the white bristle can be identified, although barely visible. The features in Figures 3(a), (c), and (e) show perfect matching with the features in Figures 3(b), (d), and (f). Figure 3(h) shows the 3D-rendering without the agarose surface of the OCT data, encoded in grayscale. The red overlay of the ncPAI reconstruction highlights the perfect agreement of the two measurements.

For the photoacoustic reconstruction, the full width at half maximum (FWHM) of the black bristle was measured to be 130 µm in lateral direction. This fits to the nominal diameter of 150 µm within the measurement uncertainty, given by the voxel distance in lateral direction of the reconstruction grid of 47 µm. In axial direction, the grid was chosen finer, with a voxel distance of 14 µm. The FWHM in axial direction was measured to be 85 to 90 µm. Possibly, not the whole bristle, but only the upper surface had been excited, resulting in an elliptical shape in the reconstruction. In OCT, the lateral dimension of the black bristle was found to be 150 µm, fitting to the nominal diameter. In axial direction, a diameter of 165 to 190 µm was measured. Whereas the lateral dimensions correspond to the true geometrical values, the axial dimension represents the optical path length. The latter one corresponds to the geometrical value stretched by the refractive index of agarose (*n*_1_) and hair (*n*_2_), respectively. Thus, the axial diameter of the hair is stretched by the factor *n*_2_, which is about 1.55.^37^ This would stretch the geometric diameter of 150 µm to an optic diameter of 232 nm. To match the PAI and the OCT image, the OCT image has been rescaled with respect to the refractive index in agarose, which is in first order approximation identical with the refractive index of water with *n*_1_ = 1.33. This rescales the effectively imaged diameter to a value of 174 µm, which agrees with the measured value mentioned above.

### Two-Dimensional Measurement on a Maple Fruit Phantom

**Figure 4.**
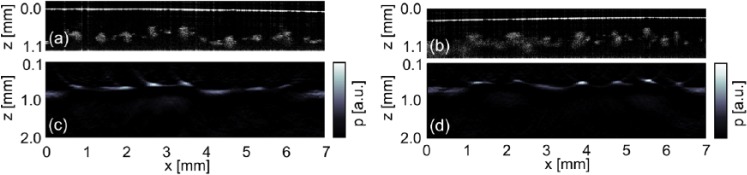
Skeleton samara of a maple tree imaged by OCT (a, b) and by ncPAI (c, d). OCT = optical coherence tomography; PAI = photoacoustic imaging.

For many applications, a B-scan is sufficient to estimate the depths of features, for example, blood vessels, from 2D images. Two-dimensional imaging was performed on a samara/agarose phantom. The slightly decomposed fruit of a maple tree was found near the research institute and embedded into agarose. Its veins range in size from 100 µm to 300 µm, comparable with the sizes of blood vessel as found in a human palm.^2^ Two B-scans were acquired with OCT and ncPAI. Figures 4(a) and (c) show a region of the skeleton samara, where only the veins of the skeleton are left. In contrast, Figures 4(b) and (d) show a region at the left-hand side of the image, where some papery tissue between the veins is still intact. The B-scan images for OCT clearly show the cross-sections of the veins. The papery tissue in between is highly scattering and does not provide a sharp interface to the agarose. The OCT images are in accordance with the ncPAI images and the location of the features match.

## Discussion and Outlook

In the “Optical Setup: Remote PAI and OCT” section, the ncPAI setup was presented. Implementation of the interferometer in a fiber-optic network allows the straightforward integration of other fiber-based optical modalities, if their wavelength regions are compatible. For ncPAI, a wavelength of 1550 nm was used for two reasons. First, high-quality low-bandwidth laser sources are available and, second, this wavelength allows amplification of light by optical amplification, that is, by an EDFA. In addition, in this wavelength region, high-quality fiber-optic components are available at relatively low costs. As a result, Corning SMF-28e+ fibers were used for the setup, which are specified for wavelengths above 1260 nm and exhibit a region of zero wavelength dispersion between 1304 nm and 1324 nm. The quality of OCT images is highly sensitive to dispersion. We therefore assembled the OCT system using the wavelength region around 1310 nm. In addition, OCT systems at 1310 nm are commonly used in various biomedical applications, including tissue imaging.

In this work, the OCT and ncPAI data were acquired sequentially. Alternatively, OCT and ncPAI data could be acquired simultaneously, by triggering of PAI data acquisition and of the OCT camera by the excitation laser. However, as OCT imaging is much faster than ncPAI, sequential scanning has no serious influence on the total scanning time. However, triggering the OCT camera with the laser pulses would reduce the resolution of the OCT B-scan. In this work, we acquired ultrasonic signals at 150 points for each B- scan, while the OCT B-scan consisted of 1040 individual A-scans. Sequential scanning allows an individual focusing for ncPAI and OCT. Usually, the focus of OCT is set beyond the surface inside the sample or the sample is tilted, to reduce local reflection of high intensity from the surface. However, ncPAI requires the focus to coincide with the sample surface, to maximize backscattering into the system. Although not demonstrated in this work, the focus could be set at different depths for ncPAI and OCT by translation of the sample or the focal lens. Implementation of this feature is one of the planned improvements of the present system.

As OCT and ncPAI use the same lens, a compromise regarding the optimal spot size has to be made. Typical spot sizes for 1310 nm OCT systems are larger than 10µm, although smaller spot sizes are possible, when trading better lateral resolution with a smaller depth of focus. In ncPAI, the spot size should be smaller than the acoustic wavelength. Thus, smaller spot sizes allow for a better photoacoustic resolution, if the detection bandwidth of the photodetector is high enough. However, for biomedical imaging, the fluence should be below the MPE. Therefore, larger spot sizes are favored, as they allow for a higher beam power. In our setup, we use a collimator with a collimated beam diameter of 7 mm and a lens with a focal distance of 75 mm. This results in a spot diameter larger than 20 µm, which permits acquisition of acoustic waves of frequencies up to 50 MHz. The frequency of 50 MHz is matched to the bandwidth of the photodetector with 45 MHz. With this setup, the expected resolution for OCT is 20 µm and 7.5 µm in lateral and axial direction, respectively. For the presented measurements, the focus had been set to the sample surface, and the sample was therefore out of focus for OCT. The lateral resolution was consequently further degraded. For ncPAI, the obtainable resolution is around 20 µm. However, as a step size of about 50 µm was chosen for the measurements, the actual resolution achieved in this paper is lower. In the present setup, the photoacoustic resolution could have been enhanced by about a factor of two by scanning of the interrogation beam with a slower velocity. Further improvements of the resolution would require a reduction of the spot size and a higher detection bandwidth. For measuring acoustic frequencies higher than 200 MHz, an excitation laser with shorter pulse lengths as the employed 4 to 6 ns has to be used. The resolution of ncPAI is also affected by the overall detection aperture (scan area).^2^ Resolution is highest in the center of the scan area and decreases with increasing imaging depth. A detailed discussion on the locally varying resolution inside the imaged volume is given in Zhang et al.^1^ For a given number of scanning points, it is essential to balance the dimension of the scanning area and the distance between the sequential points, to achieve best resolution. For imaging in larger depths, optical scattering of the excitation laser beam and acoustic attenuation will become an issue and should be taken into account for the photoacoustic reconstruction.^38-40^ According to our experience, these effects become significant for depths exceeding 5 mm. For high-resolution imaging, one may also consider the angle of incidence of the photoacoustic waves in the PA reconstruction. In ncPAI, the surface displacement rather than the pressure is measured. Waves arriving at shallow angles result in smaller displacements compared with waves impinging with steep angles. This effect can be compensated in Fourier space.^41^

As shown in the sections “Sample Preparation” and “Measurement Procedure,” the recorded OCT and ncPAI images are co-registered intrinsically in lateral direction. Co-registration in *z*-direction requires the knowledge of the refractive index and the sound velocity for OCT and the PAI reconstruction, respectively. A wrong refractive index leads to shifting of the features in *z*-direction in OCT. In the PAI reconstruction, the assumption of an incorrect sound velocity leads to displaced features. In addition, an incorrect sound velocity also leads to reconstruction artifacts, like blurring of the image, and is usually apparent. Reconstruction artifacts can also occur if the surface of the specimen is not flat but is assumed to be flat in the reconstruction. For our examined specimen the surface was approximately flat and, thus, an FSAFT algorithm was used for reconstruction, which intrinsically assumes the surface to be a flat plane. If the surface modulation is larger, other reconstruction algorithms can be used, which take the morphology of the surface into account.^42^ In this case, the OCT data can be used to identify the shape of the surface. This information can then be fed into the reconstruction algorithm.

In the “Sample Preparation” section, we presented 3D imaging. For this purpose, the interrogation beam was scanned in two dimensions. Approximately in the middle of the region, around *y* = 2 mm, backscattering was low, and thus, only weak photoacoustic signals were acquired. Usually, the output power of the PA detection laser is set in such a way that enough light is backscattered into the imaging system, but the photodetector does not saturate. In the presented measurement, the output power of the FL was about 5 mW, which is only about one-fifth of the available power. A higher output power at this position would most likely have led to a better image quality. In future, measurement of the backscattered radiant flux could allow dynamic adjusting of the laser power, to keep the light power that is guided into the ncPAI system constant. Generally, the rougher surfaces of biological samples, such as skin, exhibit higher scattering and therefore, the angle between detection beam and surface is not as critical as for the agarose phantoms. A scattering surface, however, results in waveform distortions of the reflected sample beam. This leads to problems, as mixing of a distorted sample beam with a planar reference beam is inefficient. Thus, for rough surfaces, usually self-referential interferometers, such as the two wave mixing interferometer^18,43^ or the confocal Fabry-Pérot interferometer,^44^ are used. In our approach, we use mode cleaning of the sample beam. The reflected sample beam is collected by the collimator and focused onto the single mode fiber. By sending the sample beam through the SMF, the beam is cleaned and exhibits a Gaussian profile at the output if the fiber is sufficiently long to strip off cladding modes, which usually requires only a few centimeters. The mode-cleaned sample beam can then be interfered with the reference beam that also exhibits a Gaussian profile. Mode cleaning comes at the cost of power losses that we compensate for by optical amplification using the EDFA. Depending on the speckle structure, the amount of collected light may vary from point to point. Consequently, also the modulation depth and the sensitivity of the interferometer will vary. To compensate for sensitivity fluctuations, the power of the sample beam (after the EDFA and the filter) should be monitored and the recorded signals should be normalized accordingly. This will be subject of a future improvement of the setup.

In Hochreiner et al.,^20^ we calculated the theoretical limits for the minimal detectable pressure (MDP) under typical imaging conditions to be 380 Pa for a detection bandwidth of 20 MHz and 50 Pa for a bandwidth of 5 MHz. In the current implementation, we found the MDP to be a factor of 5 to 10 higher than the theoretical predictions. We attribute this mainly to broadband noise caused by ASE of the EDFA. We found that system noise decreases with decreasing bandwidth of the filter used to remove ASE. Currently, a filter with a spectral bandwidth of 0.25 nm is used. We expect that using a filter with an even smaller bandwidth will bring the performance closer to the theoretical predictions.

## Conclusion

We presented noncontact photoacoustic and OCT imaging. Noncontact PAI was realized by an interferometric technique. Ultrasonic signals were acquired directly on the surface of a sample by an interferometer, realized in a fiber-optic network. The fiber-optic implementation allowed the addition of a SD-OCT system. The light sources were multiplexed into one fiber and the same focusing optics were used for imaging. Consequently, the obtained photoacoustic and OCT images were co-registered intrinsically in lateral direction. For 3D imaging, the interrogation beam was moved along the surface in two dimensions using a hybrid scanning technique. Fast-axis scanning was realized by means of galvanometer scanning. Perpendicular to the fast-axis, a translational stage was used for scanning along the slow-axis. We demonstrated 2D B-scan imaging of a maple fruit/agarose phantom and 3D imaging of a hair/agarose phantom.

## References

[1] Zhang E Laufer J Beard P Backward-mode multiwavelength photoacoustic scanner using a planar Fabry-Perot polymer film ultrasound sensor for high-resolution three-dimensional imaging of biological tissues. Appl Opt. 2008;47:561-77. 18239717 10.1364/ao.47.000561

[2] Zhang EZ Laufer JG Pedley RB Beard PC In vivo high-resolution 3D photoacoustic imaging of superficial vascular anatomy. Phys Med Biol. 2009;54:1035-46. 19168938 10.1088/0031-9155/54/4/014

[3] Ntziachristos V Yoo JS van Dam GM Current concepts and future perspectives on surgical optical imaging in cancer. J Biomed Opt. 2010;15:066024. 21198198 10.1117/1.3523364

[4] Rousseau G Gauthier B Blouin A Monchalin J-P Non-contact biomedical photoacoustic and ultrasound imaging. J Biomed Opt. 2012;17:061217. 22734747 10.1117/1.JBO.17.6.061217

[5] Hou Y Huang S-W Ashkenazi S Witte R O’Donnell M Thin polymer etalon arrays for high-resolution photoacoustic imaging. J Biomed Opt. 2008;13:064033. 19123679 10.1117/1.3042260 PMC2774248

[6] Grün H Berer T Burgholzer P Nuster R Paltauf G Three-dimensional photoacoustic imaging using fiber-based line detectors. J Biomed Opt. 2010;15:021306. 20459228 10.1117/1.3381186

[7] Nuster R Holotta M Kremser C Grossauer H Burgholzer P Paltauf G Photoacoustic microtomography using optical interferometric detection. J Biomed Opt. 2010;15:021307. 20459229 10.1117/1.3333547

[8] Nuster R Gruen H Reitinger B Burgholzer P Gratt S Passler K Downstream Fabry-Perot interferometer for acoustic wave monitoring in photoacoustic tomography. Opt Lett. 2011;36:981-3. 21403749 10.1364/OL.36.000981

[9] Rosenthal A Razansky D Ntziachristos V Wideband optical sensing using pulse interferometry. Opt Express. 2012;20:19016-29. 23038542 10.1364/OE.20.019016

[10] Berer T Veres IA Grün H Bauer-Marschallinger J Felbermayer K Burgholzer P Characterization of broadband fiber optic line detectors for photoacoustic tomography. J Biophotonics. 2012;5:518-28. 22371304 10.1002/jbio.201100110

[11] Nuster R Slezak P Paltauf G High resolution three-dimensional photoacoustic tomography with CCD-camera based ultrasound detection. Biomed Opt Express. 2014;5:2635-47. 25136491 10.1364/BOE.5.002635 PMC4132994

[12] Payne BP Venugopalan V Mikić BB Nishioka NS Optoacoustic tomography using time-resolved interferometric detection of surface displacement. J Biomed Opt. 2003;8:273-80. 12683854 10.1117/1.1559727

[13] Carp SA Guerra A III Duque SQJ Venugopalan V Optoacoustic imaging using interferometric measurement of surface displacement. Appl Phys Lett. 2004;85:5772-4.

[14] Carp SA Venugopalan V Optoacoustic imaging based on the interferometric measurement of surface displacement. J Biomed Opt. 2007;12:064001. 18163817 10.1117/1.2812665

[15] Berer T Hochreiner A Zamiri S Burgholzer P Remote photoacoustic imaging on solid material using a two-wave mixing interferometer. Opt Lett. 2010;35:4151-3. 21165120 10.1364/OL.35.004151

[16] Wang Y Li C Wang RK Non-contact photoacoustic imaging achieved by using a low-coherence interferometer as the acoustic detector. Opt Lett. 2011;36:3975-7. 22002357 10.1364/OL.36.003975

[17] Rousseau G Blouin A Monchalin J-P Non-contact photoacoustic tomography and ultrasonography for tissue imaging. Biomed Opt Express. 2011;3:16-25. 22254164 10.1364/BOE.3.000016 PMC3255333

[18] Hochreiner A Berer T Grün H Leitner M Burgholzer P Photoacoustic imaging using an adaptive interferometer with a photorefractive crystal. J Biophotonics. 2012;5:508-17. 22354686 10.1002/jbio.201100111

[19] Park SJ Eom J Kim YH Lee CS Lee BH Non-contact photoacoustic imaging based on all-fiber heterodyne interferometer. Opt Lett. 2014;39:4903-6. 25121904 10.1364/OL.39.004903

[20] Hochreiner A Bauer-Marschallinger J Burgholzer P Jakoby B Berer T Non-contact photoacoustic imaging using a fiber based interferometer with optical amplification. Biomed Opt Express. 2013;4:2322-31. 24298397 10.1364/BOE.4.002322 PMC3829530

[21] Huang D Swanson EA Lin CP Schuman JS Stinson WG Chang W Optical coherence tomography. Science. 1991;254:1178-81. 1957169 10.1126/science.1957169 PMC4638169

[22] Bouma BE Tearney GJ Bouma B, herausgeber. Handbook of Optical Coherence Tomography. New York: Marcel Dekker; 2002.

[23] Stifter D Beyond biomedicine: a review of alternative applications and developments for optical coherence tomography. Appl Phys B. 2007;88:337-57.

[24] Shakhov AV Terentjeva AB Kamensky VA Snopova LB Gelikonov VM Feldchtein FI Optical coherence tomography monitoring for laser surgery of laryngeal carcinoma. J Surg Oncol. 2001;77:253-8. 11473374 10.1002/jso.1105

[25] Boppart SA Brezinski ME Pitris C Fujimoto JG Optical coherence tomography for neurosurgical imaging of human intracortical melanoma. Neurosurgery. 1998;43:834-41. 9766311 10.1097/00006123-199810000-00068

[26] Zhang EZ Povazay B Laufer J Alex A Hofer B Pedley B Multimodal photoacoustic and optical coherence tomography scanner using an all optical detection scheme for 3D morphological skin imaging. Biomed Opt Express. 2011;2:2202-15. 21833358 10.1364/BOE.2.002202 PMC3149519

[27] Li L Maslov K Ku G Wang LV Three-dimensional combined photoacoustic and optical coherence microscopy for in vivo microcirculation studies. Opt Express. 2009;17:16450-5. 19770860 10.1364/OE.17.016450 PMC2855548

[28] Jiao S Jiang M Hu J Fawzi A Zhou Q Shung KK Photoacoustic ophthalmoscopy for in vivo retinal imaging. Opt Express. 2010;18:3967-72. 20389409 10.1364/OE.18.003967 PMC2864517

[29] Liu T Wei Q Wang J Jiao S Zhang HF Combined photoacoustic microscopy and optical coherence tomography can measure metabolic rate of oxygen. Biomed Opt Express. 2011;2:1359-65. 21559147 10.1364/BOE.2.001359 PMC3087592

[30] Yang Y Li X Wang T Kumavor PD Aguirre A Shung KK Integrated optical coherence tomography, ultrasound and photoacoustic imaging for ovarian tissue characterization. Biomed Opt Express. 2011;2:2551-61. 21991547 10.1364/BOE.2.002551 PMC3184864

[31] Zhang X Zhang HF Jiao S Optical coherence photoacoustic microscopy: accomplishing optical coherence tomography and photoacoustic microscopy with a single light source. J Biomed Opt. 2012;17:030502. 22502553 10.1117/1.JBO.17.3.030502 PMC3380948

[32] Liu T Wei Q Song W Burke JM Jiao S Zhang HF Near-infrared light photoacoustic ophthalmoscopy. Biomed Opt Express. 2012;3:792-9. 22574266 10.1364/BOE.3.000792 PMC3345807

[33] Berer T Leiss-Holzinger E Hochreiner A Bauer-Marschallinger J Leitner M Buchsbaum A Multimodal non-contact photoacoustic and OCT imaging using a fiber based approach. Proc SPIE. 2014;8943:894345.

[34] Berer T Leiss-Holzinger E Hochreiner A Bauer-Marschallinger J Buchsbaum A Multimodal non-contact photoacoustic and optical coherence tomography imaging using wavelength-division multiplexing. J Biomed Opt. 2015;20 (forthcoming). 10.1117/1.JBO.20.4.046013 25919425

[35] Bauer-Marschallinger J Felbermayer K Hochreiner A Grün H Paltauf G Burgholzer P Low-cost parallelization of optical fiber based detectors for photoacoustic imaging. Proc SPIE. 2013;8581:85812M.

[36] Busse LJ Three-dimensional imaging using a frequency-domain synthetic aperture focusing technique. IEEE Trans Ultrason Ferroelectr Freq Control. 1992;39:174-9. 18263134 10.1109/58.139112

[37] Greenwell M Willner A Kirk P Human hair studies: III. Refractive index of crown hair. J Crim Law Criminol. 1941;31:746-52.

[38] Lutzweiler C Razansky D Optoacoustic imaging and tomography: reconstruction approaches and outstanding challenges in image performance and quantification. Sensors. 2013;13:7345-84. 23736854 10.3390/s130607345 PMC3715274

[39] Bauer-Marschallinger J Berer T Grun H Roitner H Reitinger B Burgholzer P Broadband high-frequency measurement of ultrasonic attenuation of tissues and liquids. IEEE Trans Ultrason Ferroelectr Freq Control. 2012;59:2631-45. 23221212 10.1109/TUFFC.2012.2504

[40] Roitner H Bauer-Marschallinger J Berer T Burgholzer P Experimental evaluation of time domain models for ultrasound attenuation losses in photoacoustic imaging. J Acoust Soc Am. 2012;131:3763-74. 22559352 10.1121/1.3699194

[41] Berer T Hochreiner A Roitner H Burgholzer P Reconstruction algorithms for remote photoacoustic imaging. IEEE Int Ultrason Symp. 2012;2012:2317-20.

[42] Berer T Hochreiner A Roitner H Grün H Burgholzer P Remote photoacoustic imaging on non-flat surfaces and appropriate reconstruction algorithms. Proc SPIE. 2013;8581:858134.

[43] Blouin A Monchalin J-P Detection of ultrasonic motion of a scattering surface by two-wave mixing in a photorefractive GaAs crystal. Appl Phys Lett. 1994;65:932-4.

[44] Shan Q Chen CM Dewhurst RJ A conjugate optical confocal Fabry-Perot interferometer for enhanced ultrasound detection. Meas Sci Technol. 1995;6:921-8.

